# *KMT2B*-related disorders in Austria: clinical features and long-term outcome after deep brain stimulation

**DOI:** 10.3389/fneur.2026.1727854

**Published:** 2026-02-09

**Authors:** Elisabetta Indelicato, Sandy Siegert, Agnes Langer, Anna Hussl, Philipp Mahlknecht, Katherina Mair, Anna Eberl, Michael Zech, Wolfgang M. Schmidt, Sylvia Boesch

**Affiliations:** 1 Center for Rare Movement Disorders Innsbruck, Department of Neurology, Medical University Innsbruck, Innsbruck, Austria; 2 Center for Rare Neurological Disorders with a Focus on Rare Pediatric Movement Disorders, Division of Pediatric Pulmonology, Allergology and Endocrinology, Department of Pediatrics and Adolescent Medicine, Medical University of Vienna, Vienna, Austria; 3 Department of Neurology, Medical University of Vienna, Vienna, Austria; 4 Institute of Neurogenomics, Helmholtz Munich, Neuherberg, Germany; 5 Institute of Human Genetics, Technical University of Munich, School of Medicine, Munich, Germany; 6 Institute for Advanced Study, Technical University of Munich, Garching, Germany; 7 Neuromuscular Research Department, Center for Anatomy and Cell Biology, Medical University of Vienna, Vienna, Austria

**Keywords:** deep brain stimulation, dyskinetic cerebral palsy, dystonia, *KMT2B*, long-term outcome

## Abstract

**Introduction:**

Since its initial description in 2016, *DYT-KMT2B* has emerged as one of the most common genetic causes of early-onset dystonia. Subsequent reports have expanded its phenotypic spectrum, frequently including neurodevelopmental features. Deep brain stimulation of the globus pallidus internus (GPi DBS) has become a therapeutic mainstay; however, most published data are based on single cases or short-term observations, and long-term outcomes remain poorly characterized.

**Methods:**

We report the clinical course and response to GPi DBS in nine patients with *KMT2B* variants prospectively followed at two Austrian national reference centers for rare movement disorders. Long-term follow-up data (range: 5–20 years) were available for six patients. Clinical features and treatment outcomes were compared with previously published cohorts.

**Results:**

Non-motor features such as developmental delay, intellectual disability, and epilepsy were more frequent in our cohort than in earlier reports. All patients developed generalized dystonia and bulbar involvement over time, emphasizing the progressive nature of the disease. Despite secondary symptom worsening during long-term follow-up, GPi DBS preserved ambulation in three patients and enabled sustained recovery of walking ability in two, maintaining functional independence. Surgical correction of foot deformities further supported mobility. Notably, *KMT2B* variants were identified upon genetic re-evaluation in two patients previously diagnosed with dyskinetic cerebral palsy.

**Discussion:**

Our long-term data underscore the progressive but heterogeneous course of *DYT-KMT2B*. GPi DBS offers durable clinical benefits, particularly when initiated before loss of ambulation. Early surgical intervention and multidisciplinary management are essential to optimize long-term outcomes.

## Introduction

*KMT2B* encodes the histone-lysine N-methyltransferase 2B, an epigenetic writer that methylates lysine residue K4 on histone H3 (1). H3K4 methylation by *KMT2B* is associated with active transcription and plays an essential role in the normal development and maturation of brain circuits involved in motor control (2, 3). *KMT2B* was associated for the first time with a monogenic disorder in 2016 (4), when Zech and colleagues identified loss of function variants in patients with childhood-onset dystonia, frequently accompanied by non-motor features, in few Austrian and German families.

*KMT2B*-related dystonia, also known as DYT-*KMT2B* (5) (MIM # 617284) typically starts in the lower limbs and usually progresses to generalized dystonia with prominent involvement of the craniocervical muscles (4, 6). Affected individuals become symptomatic during infancy or early childhood, with rare cases of symptom onset up to the age of 69 years with isolated blepharospasm (7). Dystonia may be accompanied by spasticity, muscular hypotonia and other movement disorders including tremor, myoclonus, parkinsonism and chorea (8). Along the movement disorder phenotype, non-motor features typical of neurodevelopmental syndromes are also frequent in DYT-*KMT2B*. Indeed, approximately 26% of patients display a global developmental delay and cognitive impairment is a frequent issue (~42%) (8). Psychiatric symptoms such as depression or anxiety are also frequently encountered (8). Concerning other non-motor-features, up to 63% of the patients may present dysmorphic traits (8). Short stature and microcephaly are present in around 32 and 23% of cases (8). Interestingly, cumulative reports highlighted that in up to 7% of families, *KMT2B* variants result in a neurodevelopmental phenotype without evidence of dystonia (also known as “Intellectual developmental disorder, autosomal dominant 68,” MIM #619934) (8).

The large majority of *KMT2B* disease causing variants arise *de novo* (1), but a familial transmission in an autosomal dominant fashion may be found in ca. 16% of the cases (8). Remarkably, there is a broad spectrum of different phenotypes even within families (9). When the variants are transmitted within a family, parents tend to have a milder phenotype, later onset, and a non-generalized type of dystonia. Interestingly, in 42% of the inherited *KMT2B* cases, a neurodevelopmental phenotype was observed in the transmitting parent, whereas the offspring had a DYT-*KMT2B* presentation (8). Asymptomatic carriers have been also identified, with the caveat that subtle neurological features may underreported. The disease mechanisms underlying the variable expressivity of the dystonic phenotype remain to be elucidated. On one hand, different variant types are associated with different degrees of methylation alterations, a marker which in turn correlate with the severity of phenotypic manifestations (10). On the other hand, additional genetic, epigenetic, and environmental factors may play a role.

Cumulative reports showed that bilateral deep brain stimulation (DBS) of the globus pallidus internus (GPi) may be beneficial in the treatment of dystonia and allows to preserve or regain patients' mobility (1). A mean motor improvement of 49.2% in the short term has been described (8), but DBS response is heterogeneous and variable in the long term. Despite initial improvement, worsening of gait may occur in the follow-up after DBS. In a multicenter study by Cif and colleagues, all individuals treated with DBS experienced loss of ambulation during the follow-up (9). Up to date, only few long-term follow-ups (of up to 22 years) have been described among the cumulative literature on GPi DBS in *KMT2B*-related disorders (9, 11).

In the present paper, we describe the clinical features and the long-term outcome of an Austrian cohort of nine patients with *KMT2B*-related disorders followed at two specialized national reference centers for adult and pediatric movement disorders. All patients underwent GPi DBS. We describe the long-term data of six patients, including two index patients of the initial gene discovery cohort from Zech et al. (4), providing valuable information that increases our understanding of the natural history of *KMT2B*-related disorders.

## Patients and methods

Nine patients with *KMT2B* variants were recruited and prospectively followed at two Austrian national reference centers for rare movement disorders with both adult and pediatric expertise (Patients 1I to 5I in Innsbruck and patients 1V to 4V in Vienna). Disease history from clinical charts was then retrospectively collected for the present report. Causal *KMT2B* variants were detected via trio or singleton exome sequencing with consecutive carrier analysis in the parents. All reported variants were classified as likely pathogenic or pathogenic according to the American College of Medical Genetics and Genomics (ACMG) criteria (12). When genomic data alone did not permit clear pathogenicity classification, episignature analysis was applied, as described in (13), to aid variant interpretation. Variants are annotated according to the transcript NM_014727.3. We looked for the frequency of each variant in the latest update of gnomAD (v4.1.0). All patients underwent an extensive work-up before DBS, including formal neuropsychological testing and/or neurodevelopmental assessment. Comprehensive neuropsychological assessment included tests evaluating intelligence, memory, attention, language, executive functions, and processing speed. Whenever available, we reported the scores of the motor and disability subscales of the Burke-Fahn-Marsden Dystonia Rating Scale (BFMDRS-M and -D) and the Frenchay dysarthria scores obtained from the speech therapists' examinations. Follow-ups were performed 3 months after surgery and later in regular intervals, at least yearly. Adaptation of the DBS settings were undertaken whenever appropriate according to the clinical symptoms. Clinical data from this Austrian cohort was then compared with those of the literature, as provided by up to its latest update of May 2025 (8). Written informed consent for the publication was provided by each of the participants and/or their legal guardians. Patients from Innsbruck are followed within an ongoing registry study on rare movement disorders, while patients from Vienna are enrolled in the Gepestim registry (14). Both studies were approved by the respective local institutional review boards.

## Findings

**Figure 1:**
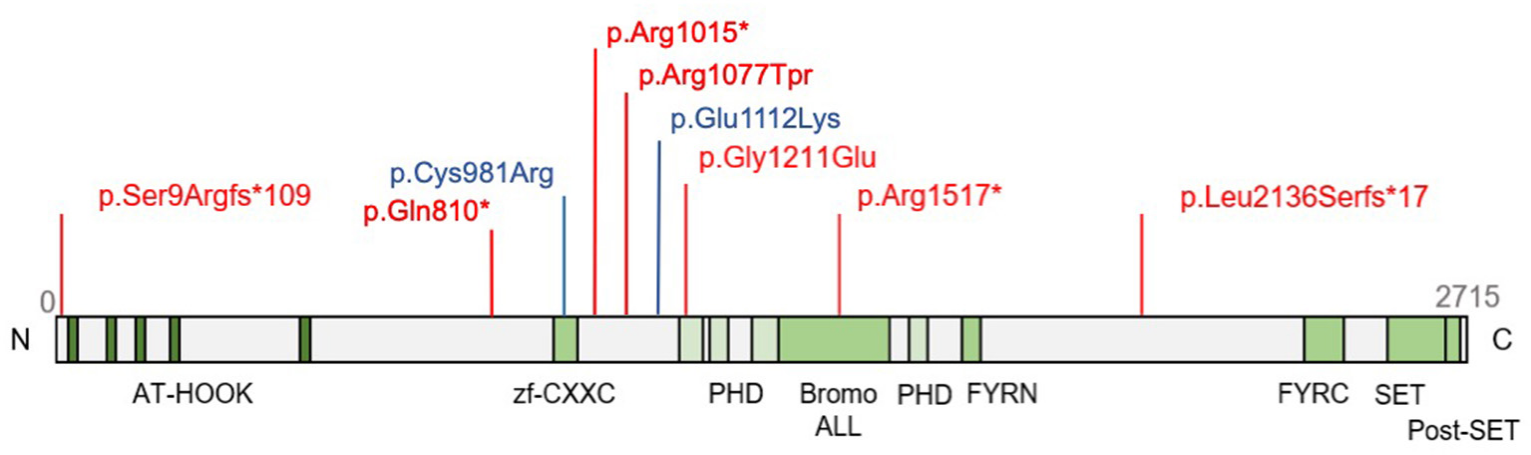
*KMT2B* variants detected in the present Austrian cohort. Variants presenting as cerebral palsy mimic are highlighted in blue.

We provide first a detailed description of the clinical course and therapy response of each patient and thereafter a comparison with those described up to date in the literature (8). The variants found in our cohort are summarized in Figure 1. Apart from the variants p.Arg1015^*^ (Patient 1V) and p.Ser9Argfs^*^109 (Patient 3I), reported by other independent submitter in ClinVar (Access September 30, 2025), all other variants were private, not reported in other families in clinical databases nor in the literature. None of the variant appears in the latest update of gnomAD v4.1.0. For a summary of the clinico-genetic characteristics of our cohort see also Table 1.

**Table 1:** Clinico-genetic characteristics of Austrian patients with DYT-*KMT2B*.

**Patient nr**.	***Patient 1I***	***Patient 2I***	***Patient 3I***	***Patient 4I***	***Patient 5I***	***Patient 1V***	***Patient 2V***	***Patient 3V***	***Patient 4V***
Variant (c.DNA, protein)	c.6406delC p.Leu2136Serfs^*^17	c.4549C>T p.Arg1517^*^	c.17_23dup p.Ser9Argfs^*^109	c.3632G>A p.Gly1211Glu	c.3334G>A p.Glu1112Lys	c.3043C>T p.Arg1015^*^	c.3229C>T pArg1077Trp	c.2428C>T p.Gln810^*^	c.2941T>C p.Cys981Arg
Inheritance	*de novo*	*de novo*	*de novo*	*de novo*	*de novo*	*de novo*	*Paternally inherited*	*Paternally inherited*	*de novo*
Already reported (reference)	*Yes*, (4)	*Yes*, (15)	*Yes*, (15)	*Yes*, (15)	*No*	*No*	*Yes*, (13)	*Yes*, (4)	*No*
**Dystonia**	Yes	Yes	Yes	Yes	Yes	Yes	Yes	Yes	Yes
Age at onset	7	6	10	7	1	6	8	6	2
Site of onset	Lower limb	Lower and upper limbs	Lower and upper limbs	Upper limb	Lower and upper limbs	Left lower limb	Right lower limb	Left lower limb	Lower limbs
Current dystonia distribution	Generalized	Generalized	Generalized	Generalized	Generalized	Generalized	Generalized	Generalized	Generalized
Type of bulbar involvement	Oromandibular dystonia	Oromandibular dystonia with tongue involvement, laryngeal dystonia (stridor)	Oromandibular dystonia	Oromandibular dystonia with tongue involvement	Oromandibular dystonia with tongue involvement	Tongue dystonia	Oromandibular dystonia with tongue involvement	Oromandibular dystonia with tongue involvement	Oromandibular dystonia with tongue involvement, laryngeal dystonia (dysphonia)
Age at onset of speech impairment	36	8	(Onset not documented, present at the age of 11)	(Onset not documented, present at the age of 20)	(Onset not documented, present at the age of 21)	9	14	6	6
**Additional features**	Yes	Yes	Yes	Yes	Yes	Yes	Yes	Yes	Yes
Intellectual disability	No	No	Learning disability	No	Circumscribed cognitive deficits, but normal IQ	Learning disability	No	Mild intellectual disability	Learning disability
Facial dysmorphism	No	Yes	No	No	Yes	Yes	Yes	Yes	No
Microcephaly	No	Yes	Yes	No	Yes	Yes	Yes	Yes	Yes
Short stature	No	No	Yes	Yes	No	No	Yes	Yes	Yes
Eye disorders	No	No	No	No	No	Optic hypotrophy	Strabismus	Strabismus	Exophoria
Epilepsy	No	No	No	No	No	Yes	Yes	No	No
Foot deformities	Pes equinovarus	No	No	No	No	Pes equinovarus	No	Pes equinovarus	No
Brain MRI	Unremarkable	Unremarkable	Neurinomas, otherwise unremarkable	Unremarkable	Unremarkable	Unremarkable	Unremarkable	Unremarkable	Unremarkable
Treatment	GPi-DBS Botulinum toxin injections Trihexyphenidyl	GPi-DBS	GPi-DBS Levodopa Gabapentin	GPi-DBS Levodopa Botulinum toxin Oxazepam Trazodone Dronabinol	GPi-DBS Trihexyphenidyl sertraline	GPi-DBS Trihexyphenidyl	GPi-DBS Levodopa Trihexyphenidyl gabapentin	GPi-DBS Botulinum toxin injections Trihexyphenidyl gabapentin	GPi-DBS Baclofen Gabapentin
Follow-up duration after DBS (years)	17	20	5	14	No follow-up yet	3	2	7	6
**BFMDRS-M**									
Pre DBS	n.a.	n.a.	21	n.a.	83	37	64	37.5	73
After DBS^*^	n.a.	n.a.	12	n.a.	64	19.5	48.5	24	50
Last FU	56 (16-year FU)	70 (20-year FU)	22 (4-year FU)	n.a.		31 (3-year FU)	58 (2-year FU)	38 (7-year-FU)	42 (6-years-FU)
**BFMDRS-D**									
Pre DBS	n.a.	n.a.	21	n.a.	83	9	13	25	25
After DBS^*^	n.a.	n.a.	12	n.a.	64	n.a.	13	7	15
Last FU	n.a.	n.a.	n.a.	n.a.		9 (3-year FU)	15 (2-year FU)	13 (7-year-FU)	n.a.
**Frenchay dysarthria**									
Pre DBS	n.a.	n.a.	n.a.	n.a.	n.a.	171/224	145/224	102/224	n.a.
1-year FU						177/224	148/224	163/224	69/224
Overall effect of DBS	Improvement; stable in the FU	Improvement; stable in the FU	Improvement; secondary worsening in the FU	Improvement; secondary worsening in the FU	Setting effect after implantation; no FU yet.	Improvement; secondary worsening in the FU	Improvement; secondary worsening in the FU	Improvement; secondary worsening in the FU	Improvement; stable in the FU

n.a., not available/not applicable; GPi, globus pallidus internus; DBS, deep brain stimulation; FU, follow-up; BFMDRS-M, Burke-Fahn-Marsden dystonia rating scale motor subscale; BFMDRS-D, Burke-Fahn-Marsden dystonia rating scale disability subscale.

^*^Best BFMDRS score post-operative from 3 to 12 months after DBS.

### Patient 1I

This patient is a 40-year-old woman, who carries the *KMT2B* variant c.6406del, p.(Leu2136Serfs^*^17). She corresponds to the index patient (F1-II-5) of the discovery cohort from 2016 (4). After an unremarkable early development, dystonia appeared for the first time at the age of seven, with dystonic inner rotation of the left foot. In the following years, the disease generalized with a classical caudal-rostral progression, first affecting the right upper limb (writer's cramp) and successively the trunk and the neck.

At her first referral in Innsbruck, at the age of 23, she was wheelchair bound. A brain MRI performed at that time was unremarkable. A previous trial with trihexyphenidyl was soon discontinued due to side effects.

She underwent GPi DBS in Innsbruck at the age of 23. GPi DBS led to significant improvement in mobility. Over the following years, she regained the ability to walk and write. Residual symptoms (cervical dystonia) were controlled with botulinum toxin injections. Because of persisting pes equinovarus deformity of the left foot, she underwent a clubfoot surgery, which further improved her ability to walk. Currently, 17 years after DBS implantation, dystonia in this patient remained well-controlled, allowing her to walk longer distances with sticks and live independently. However, in the last 4 years, mild speech problems, consistent with mild oromandibular dystonia, appeared, as well as a worsening of the right-side dystonia.

Family studies showed that neither her parents nor her four older, healthy siblings carried the variant.

### Patient 2I

This patient is a 39-year-old Austrian woman with generalized dystonia carrying the variant c.4549C>T (p.Arg1517^*^) in *KMT2B* [reported, without clinical data, in (15)]. She had a normal motor and intellectual development until the age of five. Dystonic movements first manifested in her left leg and her right arm at the age of six, with subsequent deterioration in speech and swallowing. Around the age of eight, she became anarthric. At the time of the first presentation in Innsbruck, at the age of 18, she had a severe generalized dystonia and was wheelchair bound. At the age of 19 years, she received GPi DBS. After DBS she regained the ability to walk assisted by a wheeled walker. She is however still anarthric and shows signs of mild rest dystonia in her trunk and dystonic foot inversion bilaterally.

Trio analysis confirmed a *de novo* occurrence of the p.Arg1517Ter variant.

### Patient 3I

Patient 3I is a 16-year-old male with generalized dystonia. Trio analysis detected the *de novo* variant c.17_23dup (p.Ser9Argfs^*^109) in *KMT2B* in this patient [reported, without clinical data, in (15)]. In the childhood, a combined circumscribed developmental disorder (articulation, fine/coarse/graphomotor skills) was observed. Cognitive performance level was below-average. He had a short stature, was underweight and had a mild microcephaly. Dystonia appeared first at the age of 10 years, affecting his left foot and the right arm (writer's cramp). Progressively, he started struggling with balance problems. A trial with levodopa resulted in improvement of symptoms. Around 1 year later, at the age of 11 years, he was referred for DBS evaluation. He displayed generalized dystonia with blepharospasm and oromandibular involvement, involvement of both upper limbs and of the left lower limb. Brain MRI showed small neurinomas at the level of foramen magnum, otherwise no alteration of the brain parenchyma. He received GPi DBS at the age of 11. A maximal improvement of the dystonic symptoms was reached approximately 1 year after DBS (43% improvement in BFMDRS-M). Further on the dystonic symptoms in the upper and lower left limbs worsened again, but he remains independent and could engage in a job training at the age of 16.

### Patient 4I

This patient was an Austrian man, carrying a *de novo* c.3632G>A (p.Gly1211Glu) missense variant in *KMT2B* [reported, without clinical data, in (15)]. After an unremarkable motor development, writer's cramp appeared for the first time at the age of seven, followed by progressive regression of fine motor skills according to the parents. The disease progressed, to generalized dystonia. Furthermore, he had a short stature. He showed no signs of intellectual impairment. Treatment with levodopa results only in minimal improvement. A trial with trihexyphenidyl was discontinued because of side effects.

GPi DBS was implanted when he was 20. At that time, the patient displayed generalized dystonia with involvement of the oromandibular region and of the tongue. He could walk without devices. Preoperative brain MRI was unremarkable.

He initially experienced an improvement after DBS, which concerned both the cervical and limb dystonia. However, cervical dystonia reemerged, already in the second year of follow-up, with a marked retrocollis that was not satisfactorily controlled by additional botulinum toxin injections. The extensor digitorum brevis test was negative. An additional deterioration of gait and speech was observed in the subsequent years. Upon switching off the stimulation, there was slight improvement in some speech and gait features. This motivated the patient to pursue the removal of DBS system which was performed 5 years after the initial implantation. However, dystonia went on worsening in the following year, thus retrospectively DBS side effects explained only part of the global worsening which had to be attributed to a progression of the disorder. Likely in an initial attempt at self-medication, the patient increased his alcohol consumption and later developed a benzodiazepine dependency. This contributed to a general deterioration driven by the worsening of dystonia. The latter led to reconsider DBS. A new GPi DBS system was implanted at the age of 26. The stimulation was followed only by a partial, non-satisfactorily, improvement. Attempts of adapting the stimulation were limited by the emergence of side effects. Upon patient's will, the stimulation was switched off. At the age of 28 years, he was wheelchair bound and anarthric. He refused a percutaneous gastrostomy tube. Progressive dysphagia led to underfeeding and recurrent aspiration pneumonia which further contributed to the general worsening and eventually shifting to a palliative care setting. He died at the age of 34 years.

### Patient 5I

This patient is a 23-year-old man from Austria. He was born premature in the 32th pregnancy week and had been admitted in the neonatal intensive care unit after birth. Dystonic features were already apparent at the age of 1 year. He was initially diagnosed with dyskinetic cerebral palsy, which was considered due to premature birth with presumed perinatal damage. In early childhood, developmental delay was also evident. Due to hypoacusis, the patient had been wearing hearing aids since his third year of life. At the age of around 20 years, he suffered twice from dystonic storms triggered by infections. This led to a clinical revaluation with genetic testing and finally detection of a *de novo* variant c.3334G>A (p.Glu1112Lys) in *KMT2B*.

At the first clinical examination in Innsbruck at the age of 21, he showed a generalized dystonia with predominant craniocervical involvement, with marked retrocollis and severe dysarthria (BFMDRS-M score = 83). Furthermore, neurological examination revealed upper motor neuron signs, including generalized hyperreflexia and bilateral extensor plantar responses. He had no relevant dysphagia. He could walk freely, but upon longer distances an increasing dystonic inner rotation of his legs led to stumbling and falling. Thus, he used a wheelchair for longer distances. He had microcephaly and slight dysmorphic features. Brain MRI was unremarkable. He was on trihexyphenidyl since early childhood. Additionally, he was taking sertraline.

Based on the genetic findings and on the recent occurrence of repeated dystonic storms, GPi DBS was recommended and recently performed at our center. Postoperatively some improvement of upper limber dystonia, interpretable as setting effects, were noticed (BFMDRS-M score = 68 five days after DBS implantation). This improvement was sustained in the follow-up 3 months after surgery (BFMDRS-M score = 64 point).

### Patient 1V

This patient is a 14-year-old boy born in Austria to non-consanguineous parents from Bulgaria and Turkey. Lower limb dystonia (dystonic inner rotation of the left foot) appeared for the first time at the age of 6 years after an unremarkable motor development. Dystonia subsequently spread to the upper limb and to the cervicocranial region, with dysarthria becoming evident by the age of nine. At the time of first referral in Vienna, at the age of 11, he had a generalized dystonia with marked upper limb hyperkinesia with choreoathetosis of the right arm, which had led to an external diagnosis of Sydenham's chorea. He was still ambulatory. A brain MRI at the age of 11 was unremarkable.

Concerning non-motor features, a delayed language development became evident already before the onset of dystonia. At the age of 11, learning disability were confirmed by means of Wechsler Tests. Furthermore, he developed generalized epilepsy in childhood. After exhibiting generalized tonic-clonic seizures at the age of 13 years, he was started on levetiracetam, and he is seizure free since then.

Genetic testing by Trio exome sequencing revealed a *de novo* c.3043C>T (p.Arg1015^*^) variant in *KMT2B*.

He underwent GPi DBS at the age of 11 years. At that time, he was still ambulatory and GPi DBS resulted in a stable clinical state with a slight improvement of fine motor skills. Currently, at the age of 14, he is still able to write and play soccer. Because of persisting pes equinovarus deformities of the left foot, he underwent a clubfoot surgery, which further stabilized his ability to walk and engage in sport activities. However, dystonia of his right arm worsened again at the last follow-up (3 years post DBS). Moreover, dysarthria worsened over time without ever being affected by DBS. Notably, he clearly experiences motor symptom worsening after a short time (< 24 h) being off stimulation. Hence, his subjective impression of the effect of DBS is still positive. Moreover, he was prescribed trihexyphenidyl before DBS, which was continued as it also shows additional beneficial effects.

### Patient 2V

This patient is a 22-year-old woman. After an initially unremarkable motor and language development, growth retardation, microcephaly and strabismus were noticed. At the age of eight she developed right foot dystonia. She was initially diagnosed with a functional disorder and received psychotherapy. From the age of 14 speech problems also emerged and, eventually, at the age of 16 she was referred to Vienna due to progressive dysarthria and generalized dystonia with prominent orofacial dyskinesia. Furthermore, the patient was diagnosed with epilepsy after several bilateral tonic-clonic seizure and detection of epileptic discharges on routine scalp EEG but is currently seizure free under levetiracetam and lamotrigine.

Genetic testing by exome sequencing revealed a novel missense variant, c.3229C>T (p.Arg1077Trp), in *KMT2B*. Targeted genetic analysis in her family showed that the variant segregates in the father and one sister. Both demonstrated mild developmental features (microcephaly, strabismus). The variant, formally of uncertain significance based on genomic data, could be reclassified as likely pathogenic based on the findings of the episignature analysis [reported in (13)]. This showed an abnormal, intermediate methylation pattern consistent with that of other disease-associated missense *KMT2B* variants.

Patient 2V was treated with trihexyphenidyl which showed a significant effect on limb dystonia. She underwent GPi DBS at the age of 17 years due to disease progression. Post-operative control, 3 months after surgery, confirmed a remarkable effect (BFMDRS-M pre surgery = 64, BFMDRS-M 3 months post-surgery 48.5) despite some gait freezing, hypomimia and speech hesitation. She developed oromandibular dystonia several months after surgery. Botulinum toxin injection was tried only once and thereafter discontinued due to pain and unsatisfactory effect. Despite persisting effect on upper limb dystonia, the dysarthria and motor disabilities progressed, and she is currently wheelchair bound. She is also going on taking trihexyphenidyl, as it still shows beneficial effects.

### Patient 3V

Patient 3V is now a 14-year-old girl. She corresponds to the patient F4-III-2 of the discovery cohort from 2016 (4). She was born to non- consanguineous parents and delivered via primary C-section due to a gemini pregnancy at 37 weeks of gestation. Her motor development was normal with free walking at the age of 13 months, but she displayed delayed language development. Motor symptoms appeared at the age of six with dystonic inward turning of the left foot and dysarthria. She progressed to generalized dystonia within the following year and became wheelchair bound. At the time of the first referral at the age of six, microcephaly, strabismus and mild intellectual disability were also evident. A trial of levodopa had no effect on dystonia. Genetic testing revealed a c.2428C>T (p.Gln810^*^) variant in *KMT2B*, which she had inherited from the father, who suffered from focal dystonia (right arm). Due to the progressive course and the detection of the *KMT2B* variant she underwent GPi DBS at the age of seven. After DBS she initially regained mobility and was again able to walk again with a cane or even freely. Dysarthria improved mildly. Two years after limb dystonia progressed and trihexyphenidyl was initiated with a stabilizing effect. In the last year the child is suffering from ongoing disease progression causing again an increasing need for wheelchair. Furthermore, she recently underwent a clubfoot surgery, because of persisting pes equinovarus deformities of the left foot. Dysarthria is stable without dysphagia. The patient is microcephalic and her weight and height remain below the 3rd percentile.

### Patient 4V

The patient is a currently 26-year-old adult from Austria who was initially diagnosed with dyskinetic cerebral palsy. He was born prematurely at 26 weeks of gestation. Motor development was slightly delayed with free walking at the corrected gestational age of 15 months. His language development was delayed. He received growth hormone therapy due to delayed growth. Only at the age of two he presented with progressive gait problems caused by lower limb dystonia (left > right foot). At the age of six, dysarthria and swallowing difficulties markedly worsen. Worsening dysphagia caused severe decrease of body weight. After intermittent nasogastric tube feeding with high-calories supplemental nutrition his body weight stabilized. At the age of 12 years, trials with levodopa and trihexyphenidyl were attempted with no substantial effect. A through revaluation was performed, including brain MRI, which was unremarkable.

Due to the progressive motor impairment, the boy received GPi DBS at the age of 14. DBS was initially beneficial, with the best effect on motor function reached around 1 year after implantation. Thereafter dystonia progressed. GPi DBS had no effect on dysarthria and dysphagia. Frenchay dysarthria score increased from 1 year post DBS (69/224) to 6 years post DBS (80/224). Due to progressive dysphagia, he received a percutaneous gastrostomy tube at the age of 23. The DBS system was switched off during the surgery for tube placement, revealing a significant increase of dystonic movements, particularly in the lower limbs. Due to the marked effect of GPi DBS combined with clinical features suggestive of DYT-*KMT2B*, a data from genetic testing by exome sequencing a performed in 2013 were re-evaluated in 2022. This revealed a *de novo* c.2941T>C (p.Cys981Arg) variant in *KMT2B*, leading to the diagnosis of cerebral palsy being revised. The man is still ambulatory. A wheeled walker or a wheelchair is only needed for longer distances.

### Summary of clinical features and comparison with the literature

Abela and Kurian (8) summarized clinical features and findings, therapeutic management, and genetic characteristics of patients with variants in *KMT2B* on GeneReviews (last updated on May 22, 2025). DYT-*KMT2B* has been described in 258 patients from 229 families to date. Patient 1I is included in this review; however long-term follow-up data for this patient were not published up to date. The clinical findings from the DYT-*KMT2B* from literature as compared to those from the present cohort are summarized in Table 2. *KMT2B* variants occurred *de novo* in 84% of the cases reported in the literature and in 7 out of 9 of our patients (78%). The median age at onset of dystonia in our cohort was 6 (range 1 to 10), consistent with the literature (median onset of DYT-*KMT2B* = 6 years, range from 0 to 43 years).

**Table 2:** Clinical features of DYT-*KMT2B* in the present Austrian cohort and in the reported literature.

**Clinical findings**	***Austrian cohort (n = 9)***	***Literature casuistic (n = 258)***
Generalized dystonia	100	~68
Laryngeal dysfunction	22	~51
Other movement disorders	11	~27
Intellectual disability	56	~42
Developmental delay	56	~26
Neurobehavioral/psychiatric	56	~26
Seizures/epilepsy	22	~2
Microcephaly	56	~44
*KMT2B-*related brain MRI abnormalities	0	~27

The table reports the percentage of patients who exhibit the specified characteristic. The percentages refer to the specified cohort.

Onset in the lower limbs and a progressive course of symptoms are typical features of DYT-*KMT2B* which were consistently found in our cohort. One peculiar feature that is underreported, but was noted in all our patients, was the asymmetrical distribution of dystonia at the time of onset, which also persisted—or became even more evident (as in Patient 1V)- during progression. A generalization of dystonia symptoms has been reported in ~68% of patients from the literature within two to 11 years from the initial presentation, while all our patients developed generalized dystonia. The exact type of cranial/bulbar involvement is not always clearly reported in the literature. While laryngeal dysfunction is reported in ~51% of DYT-*KMT2B* patients, data on oromandibular and tongue involvement are less detailed. All of our patients presented oromandibular dystonia with or without tongue involvement and all presented clinically with dysarthria already in the childhood/adolescence. Dysphonia and/or stridor as a consequence of laryngeal involvement was seen in two of our patients. Dysphagia was present at different time point in three of our patients and is similarly described in ~30% of DYT-*KMT2B* patients in the literature. Dystonic storms were observed in one Austrian patient (5I). While the prevalence of this feature in DYT-*KMT2B* is not known, few reports of its possible evolution, the life-threatening status dystonicus, have been described in association with *KMT2B* variants (6, 9, 11, 16).

Intellectual disability was observed in five out of nine Austrian patients (56, vs. ~42% in the literature). Additionally, developmental delay was reported in 56% of our patients as compared to ~26% of cases in the literature. Neurobehavioral and psychiatric features were present in 40% of Austrian DYT-*KMT2B* cases and in ~26% of cases in the literature. Epilepsy was more frequent in our Austrian cohort as compared to the literature. Movement disorders, other than dystonia (ataxia, tremor, spasticity, myoclonus, chorea) are reported in several patients in the literature (~27%), but were observed in only one Austrian patient, who presented with prominent choreoathetosis of the right upper limb, concomitant to dystonia, which led to the first medical referral and initial suspicion of a primary choreatic disorder (Patient 1V). Brain MRI in ~27% of the patients with DYT-*KMT2B* in the literature showed abnormalities considered features of the disease (subtle and symmetrical hypointense lateral streaks in the external globus pallidus). We did not see any indicative MRI-abnormalities in our cohort. This may be explained by the observed age-dependency of these MRI changes which may become less prominent over time and are often not evident in adults.

Previous diagnosis of cerebral palsy was frequently reported in the literature as also seen in patients 5I and 4V. Importantly, genetic reevaluation and diagnosis of DYT-*KMT2B* in one patient diagnosed with cerebral palsy were prompt after successful DBS (Patient 4V).

### Summary of therapeutic response and comparison with the literature

Therapeutic options in DYT-*KMT2B* are limited to symptomatic treatment. Pharmacological options comprise levodopa, baclofen, gabapentin, and benzodiazepines, which are reported to have an effect, but no long-term benefit in most patients with DYT-*KMT2B*. Anticholinergic drugs appear to be the most effective, improving motor symptoms significantly in approximately 50% of patients in the literature (8). Similarly, three of our patients experienced a significant clinical response to trihexyphenidyl, which remained part of the therapy after DBS. Two patients of our cohort underwent a therapeutic trial with levodopa with mixed response.

A mean motor improvement of ~49% after DBS is reported in the literature at follow-up times of two to 16 months after surgery. With the caveat of missing BFMDRS-M scores and different evaluation time points, the improvement observed in our cohort ranged from 23% to 47%. In our cohort, DBS improved symptoms within the first months and years after implantation. After a steady course with stable symptoms, a mild or even moderate worsening of the symptoms was observed in all our patients, consistent with the progressive character of DYT-*KMT2B*. In one of the few long-term report available in the literature [(9), which reported on eight subjects with follow-up between 5 and 22 years], all patients experienced a loss of ambulation later on, despite the initial benefit of DBS on lower limb dystonia. In our cohort, three patients (1I, 2I, 3V) regained the previously lost capacity to walk after DBS. This effect was sustained in the long-term (12–20 years after DBS). Notably, in two patients (1I and 1V), surgical correction of foot deformities decisively contributed to preserve ambulation. A third patient (3V) recently underwent the same procedure. Analogous to the literature, our patients did not benefit from DBS concerning laryngeal dystonia (swallowing, inspiratory stridor). When available Frenchay dysarthria score was not affected or got even worse after DBS.

Freezing of gait as a side effect of the DBS was seen in two patients in our cohort (4I and 2V).

## Discussion

From its first description in 2016, DYT-*KMT2B* emerged as one of the most prevalent etiologies of early-onset dystonia (1). Cumulative reports have progressively highlighted a more complex phenotype with above all additional developmental features and even the possibility of a neurodevelopmental presentation without an apparent motor phenotype (9). Approximately 80 cases of patients with DYT-*KMT2B* who received DBS have been published (8) and contributed to establish it as a treatment mainstay. However, most of the available data derives from case reports with short follow-up. Long-term follow-up data are very limited (9, 11) but highly needed to enhance our understanding, improve counseling strategies and in turn patients care. Despite an average improvement approaching 50% in the first year of follow-up, a marked heterogeneity in progression and responses under DBS is seen in DYT-*KMT2B* without predictive biomarker available so far. Bearing this in mind, we provided here the experience of two Austrian reference centers, reporting on a representative, nation-wide cohort patients with long-term follow-up.

Our cohort reflects the wide clinical spectrum associated with *KMT2B* variants. Compared to the literature, non-motor features, such as developmental delay and intellectual disability, were even more frequent in our cohort. This might be due to more detailed investigations within an extensive pre-DBS work up and likely to the recruitment in specialized national centers, including a neuropediatric one, to which patients with a more complex and severe clinical presentation are referred. Epilepsy was also clearly more common in our cohort than in the available literature, indicating the need to perform EEGs early and to monitor for epilepsy-specific potentials over time. Considering the dystonic phenotype, a generalization of symptoms (~68% in the literature), as well as a bulbar involvement, were seen in all our patients, perhaps due to the longer observational period which allowed us to better appreciate the progressive nature of DYT-*KMT2B*. Long-term follow-up data (≥5 years) are available for six out of nine patients who underwent DBS. All our patients with long-term follow-up after DBS experienced a secondary worsening, however, in three of them ambulation was preserved by DBS and in two of them the recovery of ambulation was a sustained effect, which contributed to maintain functional independency. Our findings of effectiveness of DBS especially in still ambulatory patients underlines the importance of recommendation of this therapeutic option at an early stage of the disease as it might prolong ambulatory state or even prevent transition to the wheelchair-dependency. Notably, the management of foot deformities in two patients substantially contributed to preserve ambulation. This issue is also relevant as neurologists may refrain from referring or considering surgical correction of foot deformities in the setting of generalized dystonia. All these issues and concerns should be addressed during counseling of patients and their families. In particular, the possibility of secondary worsening in the context of a naturally progressive disease should be carefully discussed to avoid creating false expectations or diminishing acceptance of DBS over time, despite a clear symptomatic benefit, as may have occurred in patient 4I.

*KMT2B* variants are recurrently found in the setting of a genetic re-evaluation of patients previously diagnosed with dyskinetic cerebral palsy (17, 18). In this setting, *KMT2B* variants belong to the “actionable,” and thus relevant, genetic findings which may eventually allow e.g., counseling for DBS. This issue is showcased by two patients of our cohort who received a genetic revaluation in their adolescence/early adulthood after an initial diagnosis of dyskinetic cerebral palsy. In one case the occurrence of hyperkinetic crisis and in the second one the effectiveness of GPi DBS and the course of bulbar dysfunction were clinical clues which initially led to reconsider the diagnosis. While it is now recognized that a “dyskinetic” phenotype –along with absent evidence of perinatal injury- should raise concern for a genetic etiology of cerebral palsy, a number of young adults with this longstanding diagnosis may still escape a genetic allocation. Disease progression/evolution also does not fit the stable clinical phenotype of cerebral palsy. With this concern, hyperkinetic crisis and progressive bulbar dysfunction may provide a clue for *KMT2B* variant testing in this setting.

This study has several limitations. The first concerns the small sample size of our cohort. BFMDRS-M scores were not always available for all patients, limiting the consistency of outcome assessment. Despite the availability of detailed clinical data due to the involvement of expert centers and comprehensive pre-DBS evaluations, in the long term, standardized protocols need to be implemented and used across centers for the evaluation pre-DBS and post-DBS. In particular, dysarthria was not systematically assessed with objective tools such as the Frenchay Dysarthria Assessment or other validated rating scales. As bulbar involvement is a key feature in DYT-*KMT2B*, future studies are needed to evaluate its progression and management. Moreover, extensive neuropsychiatric evaluation before and after DBS should come into focus as we know from literature. Indeed, our report also highlights that DYT-*KMT2B* comes along with a broad neuropsychiatric symptoms' spectrum. Data about potential cognitive and behavioral impacts of GPi-DBS in dystonia is generally scarce. Few studies suggest no major effect of GPi DBS on neuropsychological and neuropsychiatric aspects (19). Detailed data for DYT-*KMT2B* is lacking, but systematic prospective assessments will be essential for DBS-counseling.

Finally, an important aspect which was also not consistently available in our patients are methylation studies. Methylation of lysine residues on histone tails represent a key dynamic chromatin modification that is essential for the maturation of brain circuits involved in motor control (3). Recently, a distinctive DNA methylation profile in peripheral blood of patients with *KMT2B* variants—referred to as an epi-signature—has been described (10). This biomarker may greatly contribute to variant interpretation, as seen in patient 2V (20). It furthermore supports several of the observed genotype–phenotype correlations in *KMT2B*-related disease. For example, the *KMT2B* missense variants p.Ala1541Val and p.Arg1078Cys, which are associated with adult-onset dystonia, produces more subtle methylation alterations compared to truncating variants typically linked to early-onset, developmental cases (7, 13). The critical role of proper H3K4 methylation dosage in human development is further underscored by the involvement of at least six *KMT2* genes in disease, despite their apparently redundant enzymatic activity (21). Notably, methylation is a dynamic, and potentially reversible or inducible, process. This aligns with the clinical observation that neural modulation via DBS can exert therapeutic benefit in certain *KMT2B* variants. Understanding the relationship between dysregulated *KMT2B* function, the degree of methylation change, and the resulting neurological phenotype may provide key insight into unresolved questions about the natural history of *KMT2B*-related disorders.

## Data Availability

The original contributions presented in the study are included in the article/Supplementary material, further inquiries can be directed to the corresponding author.
