# Supplementary material for: KMT2B-related disorders in Austria: clinical features and long-term outcome after deep brain stimulation
Source: Front Neurol. 2026 Feb 9;17:1727854. doi: 10.3389/fneur.2026.1727854 (PMC12926133; doi:10.3389/fneur.2026.1727854)
Supplement: Supplementary file 1 [file Data_Sheet_1.pdf]

Supplementary material

Available data about DBS settings and lead position are provided below:

**Patient 1I**	
Year of DBS	2008
Current DBS system	Medtronic Activa RC
Initial settings	Right GPi Contact 1-, 2.1 V, 120 µs, 130 Hz<br>Left GPi Contact 9-, 2.1 V, 120 µs, 130 Hz
Last settings	Right GPi Contact 1-, 3.9 V, 120 µs, 130 Hz<br>Left GPi Contact 9-, 2.8 V, 120 µs, 130 Hz

**Patient 2I**	
Year of DBS	2005
Current DBS system	Medtronic Activa RC
Initial settings	Right GPi Contact 0/1-, 3.2 V, 90 µs, 180 Hz<br>Left GPi Contact 4-/5-, 2.2 V, 90 µs, 180 Hz
Last settings	Right GPi Contact 1-, 3 V, 90 µs, 130 Hz<br>Left GPi Contact 8-/9-, 4.5 V, 90 µs, 130 Hz

**Patient 3I**	
Year of DBS	2020
Current DBS system	Boston Scientific - directional
Initial settings	Right GPi Contact L1-/L2-, 2.3 mA, 60 µs, 130 Hz<br>Left GPi Contact 1-/4-, 2.3 mA, 60 µs, 130 Hz
Last settings	Right GPi Contact L1-/L2, 6.2 mA, 60 µs, 130 Hz<br>Left GPi Contact 1-/4-, 5 mA, 60 µs, 130 Hz

**Patient 4I**	
Year of DBS	2006
Last DBS system	Medtronic Activa RC
Initial settings	Right GPi Contact 0/1-, 3.2 V, 120 µs, 130 Hz<br>Left GPi Contact 4-/5-, 3.2 V, 120 µs, 130 Hz
Last settings	Right GPi Contact 3-, 2.2 V, 90 µs, 100 Hz<br>Left GPi Contact 11-, 2.2 V, 90 µs, 100 Hz

**Patient 5I**	
Year of DBS	2025
Last DBS system	Boston Scientific
Initial settings	Right GPi Contact L2, 2 mA, 120 µs, 130 Hz<br>Left GPi Contact L1/L2, 1.5 mA, 90 µs, 130 Hz
Last settings	Right GPi Contact L2, 3 A, 90 µs, 130 Hz<br>Left GPi Contact L1/L2, 2 mA, 90 µs, 130 Hz

The figure below shows an exemplary electrode positioned in the posterolateral portion of the left GPi in patient 5I (provided with the kind permission of Brain Lab). Left GPi (orange) is represented in spatial relation with the optic radiation (dark yellow).

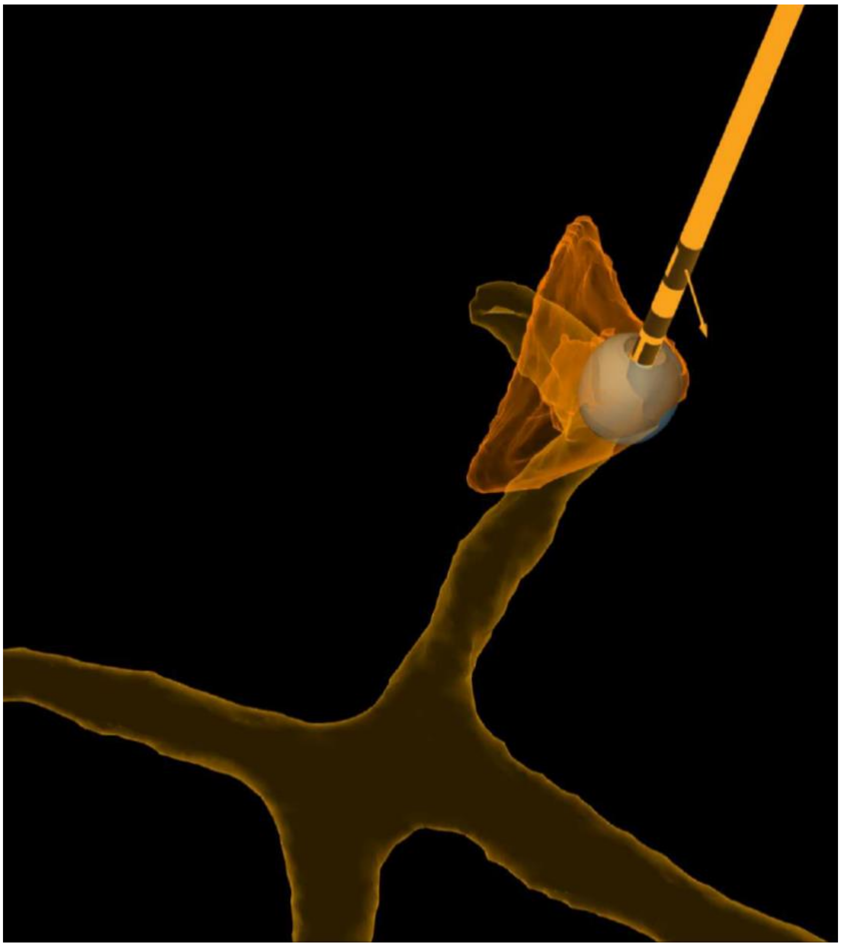

**Patient 1V**	
Year of DBS	2022
Current DBS system	SenSight Medtronic
Stereotactic coordinates (AC-PC reference in mm)	GPi L: x = 15.14; y = 4.98; z = -1.84<br>GPi R: x = 15.0; y = 4.64; z = -2.36
Initial settings	Both sides Contact 1/9, 1.5 mA, 120 μs, 130 Hz
Last settings	Both sides Contact 1/9, 2 mA, 120 μs, 130 Hz

**Patient 2V**	
Year of DBS	2021
Current DBS system	Medtronic Activa RC
Stereotactic coordinates (AC-PC reference in mm)	GPi L: X: -17.57 Y: 4.0 Z: 1.15<br>GPi R: X: 18.05 Y: 2.5 Z: 1.15
Initial settings	Both sides Contact 2/9, 1.5 V,120 μs, 130 Hz
Last settings	Both sides Contact 1/8, 3 V, 120 μs, 130 Hz

**Patient 3V**	
Year of DBS	2017
Current DBS system	Medtronic Activa RC
Stereotactic coordinates<br>(AC-PC reference in mm)	GPi L: x = -17.28; y = 3.0; z = 1.05<br>GPi R: x = 17.8; y = 3.8; z = 1.06
Initial settings	Both sides Contact 1/9, 1.5 V,120 µs, 130 Hz
Last settings	Both sides Contact 1/9, 2 V,120 µs, 130 Hz

**Patient 4V**	
Year of DBS	2014
Current DBS system	Medtronic Activa RC
Stereotactic coordinates<br>(AC-PC reference in mm)	GPi L: x = -16.5; y = 2.04; z = -1.52<br>GPi R: x = 16.5; y = 2.04; z = -1.52
Initial settings	Both sides Contact 1/9, 2.5 V,120 µs, 130 Hz
Last settings	Both sides Contact 1/9, 3 V,120 µs, 130 Hz
